# Estimating the cost of implementing district mental healthcare plans in five low- and middle-income countries: the PRIME study

**DOI:** 10.1192/bjp.bp.114.153866

**Published:** 2016-01

**Authors:** Dan Chisholm, Soumitra Burman-Roy, Abebaw Fekadu, Tasneem Kathree, Dorothy Kizza, Nagendra P. Luitel, Inge Petersen, Rahul Shidhaye, Mary De Silva, Crick Lund

**Affiliations:** **Dan Chisholm**, PhD, Department of Mental Health and Substance Abuse, World Health Organization, Geneva, Switzerland; **Soumitra Burman-Roy**, MSc, Centre for Global Mental Health, London School of Hygiene and Tropical Medicine, London, UK; **Abebaw Fekadu**, MD, Department of Psychiatry, Addis Ababa University, Ethiopia; **Tasneem Kathree**, MSc, University of KwaZulu Natal, Durban, South Africa; **Dorothy Kizza**, PhD, Butabika Mental Hospital, Kampala, Uganda; **Nagendra P. Luitel**, MA, Transcultural Psychosocial Organization (TPO) Nepal, Kathmandu, Nepal; **Inge Petersen**, PhD, University of KwaZulu Natal, Durban, South Africa; **Rahul Shidhaye**, MD, Public Health Foundation of India, Bhopal, Madhya Pradesh, Delhi, India; **Mary De Silva**, PhD, Centre for Global Mental Health, London School of Hygiene and Tropical Medicine, London, UK; **Crick Lund**, PhD, Alan J Flisher Centre for Public Mental Health, Department of Psychiatry and Mental Health, University of Cape Town, Cape Town, South Africa, and Centre for Global Mental Health, Institute of Psychiatry, Psychology and Neuroscience, King's College London, UK

## Abstract

**Background**

An essential element of mental health service scale up relates to an assessment of resource requirements and cost implications.

**Aims**

To assess the expected resource needs of scaling up services in five districts in sub-Saharan Africa and south Asia.

**Method**

The resource quantities associated with each site's specified care package were identified and subsequently costed, both at current and target levels of coverage.

**Results**

The cost of the care package at target coverage ranged from US$0.21 to 0.56 per head of population in four of the districts (in the higher-income context of South Africa, it was US$1.86). In all districts, the additional amount needed each year to reach target coverage goals after 10 years was below $0.10 per head of population.

**Conclusions**

Estimation of resource needs and costs for district-level mental health services provides relevant information concerning the financial feasibility of locally developed plans for successful scale up.

Successful scaling up of mental health services involves putting together a range of human, physical and other resource inputs in order to deliver interventions and services capable of improving mental health and related outcomes.^1^ Accordingly, an essential element of evidence-based mental health service planning and scale up relates to an assessment of what resources are required to deliver services to the population in need and meet programme goals.

In this paper, one of a series describing the development of district-level mental healthcare plans (MHCPs) in five low- and middle-income countries (LMIC) participating in the PRogramme for Improving Mental health carE (PRIME) study, the expected resource needs and costs of implementing and scaling up services in these localities are presented and examined. The work builds on earlier methods and analysis undertaken for 12 LMIC,^2^ but is pitched at the district rather than national level, is more closely based on local experiences and data, and employs a more extensive costing tool for estimation.

## Method

### Setting

In line with other papers in this supplement, analysis of resource needs and costs was carried out for five districts in sub-Saharan Africa and south Asia that span a diverse range of sociocultural, urban/rural and economic contexts, including extremely under-resourced settings (Ethiopia, Uganda), a fragile state setting (Nepal) and middle-income countries marked by high levels of socioeconomic inequality (India and South Africa). Key sociodemographic, epidemiological and economic characteristics of each study district, including estimates of population in need, are shown in Table 1.^3,4^

**Table 1:** District-level sociodemographic and epidemiological characteristics

	Sodo district (Ethiopia)	Sehore district (India)	Chitwan district (Nepal)	Kenneth Kaunda district (South Africa)	Kamuli district (Uganda)
Population, *n*	165000	1311 008	566 661	796 823	428 500

Living rurally, %	90	81	73	14	97

Literacy rate, %	22	71	70	88	62

Health spend per capita (nationally, US$)^a^	7	29	17	425	24

Cases, *n* (% population in need)^b^					
Depression	1615 (1.0)	19423 (1.5)	8126 (1.4)	15931 (2.3)	3879 (0.9)
Psychosis	481 (0.3)	4679 (0.4)	1881 (0.3)	2533 (0.4)	1114 (0.3)
Epilepsy	2342 (1.4)	–	3418 (0.6)	–	6114 (1.4)
Alcohol use disorders	8379 (5.1)	7525 (0.6)	3006 (0.5)	13851 (2.0)	19205 (4.5)

a. Source: World Health Organization (WHO) National Health Accounts database (www.who.int/nha/country/en/).

b. Source: WHO Global Burden of Disease database (www.who.int/healthinfo/global_burden_disease/en/).

### MHCPs and intervention packages

The formation and specification of each district-level MHCP is described in detail in the companion papers in this supplement.^5–9^ The specific disorders and interventions included in each country site's plan, together with current and target coverage levels, are shown in Table 2. All sites included treatment of psychosis, depression and alcohol use disorders, and three sites (Ethiopia, Nepal, Uganda) also included epilepsy treatment. The primary source for the selection of interventions was the World Health Organization (WHO) Mental Health Gap Action Programme (mhGAP) intervention guide,^10^ which was adapted according to local district needs in each PRIME country site.

**Table 2:** District-level intervention packages and coverage levels

	Sodo district (Ethiopia)		Sehore district (India)		Chitwan district (Nepal)		Kenneth Kaunda district (South Africa)		Kamuli district (Uganda)	
Disorder and interventions	Coverage, %									
	Current	Target	Current	Target	Current	Target	Current	Target	Current	Target
Depression										
Basic psychosocial treatment, advice and follow-up	5	35	5	30	1	30	5	15	5	50
Antidepressant medication	5	30	5	30	1	30	15	25	3	30
Intensive psycho- social intervention					1	15	0	5		
Psychosocial care for perinatal depression	0	5	1	30	–	–	–	–	3	30

Psychosis										
Basic psychosocial treatment, advice and follow-up	10	75	20	60	1	40	20	30	10	80
Antipsychotic medication	10	75	20	60	1	40	60	80	10	50
Intensive psycho- social intervention					1	10	0	5		

Epilepsy										
Basic psychosocial treatment, advice and follow-up	13	75	–	–	1	50	–	–	30	80
Anti-epileptic medication	13	75	–	–	10	50	–	–	20	60

Alcohol use disorder										
Identification and assessment (of new cases)	2	25	5	20	1	30	1	20	1	30
Brief interventions and follow-up	2	25	5	20	1	15	1	10	1	30
Management of alcohol withdrawal	2	25	–	–	–	–	5	20	1	20

### Assessment of resource needs and costs

The newly developed mhGAP costing tool was used to estimate resource requirements and costs of each site's intervention package (tool available from the authors on request). This is a disease-specific costing tool for short- or medium-term planning that can be used to generate forecasts of human and financial resource needs (at the national or subnational level); it is based on a methodology used to derive global cost estimates for scaling up interventions related to the Millennium Development Goals, such as HIV/AIDS, malaria and child health, as well as non-communicable diseases and mental health.^2,11^ The tool produces estimates of total and incremental costs of scaled-up provision, broken down by different mhGAP disorders, types of expenditure and year of scale up. Costs can be calculated in local currency units or in US dollars. All prices and cost values reported here are in US dollars for 2008 (no account is taken of inflation).

The cost of scaling up an intervention can be determined with reference to five key parameters:

- population (of the country, region or district);
- prevalence (of the disorder in question);
- resource quantities (needed for an intervention; for example out-patient visits, medicines);
- prices or unit costs (for each resource item or entity; for example salaries, drug prices); and
- coverage (% population in need that receives the intervention).

Multiplication of the first two parameters (population× prevalence) defines the population at risk or in need (Table 1), whereas multiplication of the final two parameters (resource use×price) provides the cost per case treated (Table 3). The remaining parameter, coverage, acts as the lever through which the total number (and associated cost) of treated cases can be estimated over a period of scaled-up service delivery (Table 2).

**Table 3:** Annual cost of World Health Organization Mental Health Gap Action Programme treatment components per average case of disorder (US$)^a^

	Sodo district (Ethiopia)	Sehore district (India)	Chitwan district (Nepal)	Kenneth Kaunda district (South Africa)	Kamuli district (Uganda)
Depression					
Basic psychosocial treatment, advice and follow-up	2.48	3.56	1.86	15.45	3.75
Antidepressant medication	33.50	11.83	29.63	55.92	11.25
Intensive psychosocial intervention	–	–	2.32	15.89	–
Psychosocial care for perinatal depression	5.05	14.48	–	–	6.71

Psychosis					
Basic psychosocial treatment, advice and follow-up	8.60	5.35	2.79	23.17	9.12
Antipsychotic medication	35.29	30.20	176.47	357.87	22.84
Intensive psychosocial intervention	–	–	6.95	14.45	–

Epilepsy					
Basic psychosocial treatment, advice and follow-up	1.24	–	1.86	–	1.52
Anti-epileptic medication	13.32	–	37.00	–	13.77

Alcohol use disorder					
Identification and assessment (of new cases)	0.40	1.14	0.60	4.95	0.61
Brief interventions and follow-up	1.24	3.56	1.86	28.05	1.90
Management of alcohol withdrawal	3.75	–	–	35.42	0.80

a. Non-specialist care only (excludes hospital-based in-patient and out-patient care); cost per average case accounts for proportion of individuals needing the intervention (for example out of 100 cases, not all individuals need diagnostic tests).

### Population in need

The population in need in each district was calculated by relating prevalence estimates for the different disorders to the total population of the district. In the absence of robust local psychiatric epidemiological surveys in all but one setting, prevalence estimates were drawn from the Global Burden of Disease database for the WHO subregion to which the country in question belonged (www.who.int/healthinfo/global_burden_disease/en/). In the South African district, the population in need for treatment of depression and alcohol use disorders was revised to cover only those with HIV and chronic non-communicable diseases such as cardiovascular disease and diabetes (which is in line with the national policy to roll-out an integrated system of care for chronic diseases).

### Resource quantities

Concerning healthcare resource profiles for each of the included interventions, the key categories of potential resource use incurred in non-specialist healthcare settings were: primary and ancillary care visits; medication; and laboratory or diagnostic tests. In addition, resources used at a higher, more specialised level of the healthcare system were considered, in particular hospital in-patient and out-patient services. Since specialist psychiatric services are largely absent at the level of the participating districts, the primary focus of the cost analysis was on non-specialised healthcare needs; specialist services were included in a secondary analysis in order to ascertain the likely range of costs that would apply if they were available and utilised. Default estimates of expected resource use were based on the treatment protocols laid out in the mhGAP intervention guide, and supplemented by information from cost-effectiveness studies of these interventions in resource-constrained settings (for an overview see Chisholm & Saxena^12^).^13^ However, since local practice or operationalisation concerning these evidence-based interventions varies appreciably, each district team reviewed and revised these profiles (see online Table DS1 for a tabulated overview of changes made).

The mhGAP costing tool also provides indicative human and other resource needs for overall mental health programme direction and administration, as well as for training and capacity building. As a result of the paucity of current mental health programme activity in districts, baseline programme-level costs were expected to be negligible. The cost of appropriate programme management, training and supervision in each district were estimated and are reported below.

### Unit costs

The price or ‘unit cost’ of healthcare services and goods come in two forms: ‘traded goods’, such as medicines or diagnostic equipment (which can be purchased on the international market); these items were valued at international price levels, adjusted for shipping and distribution costs to/within a country (default drug prices were taken from the International Drug Price Indicator Guide http://erc.msh.org/dmpguide/). ‘Non-traded’ goods included local personnel, in-patient and out-patient care, per diems for training, consumables, building costs and utilities (which are largely generated and used up within national boundaries); for these items, country-specific predicted costs were entered, using the WHO CHOICE costing database (www.who.int/choice/costs). Only a small proportion of the unit cost of a healthcare visit is for capital (a fixed cost), so the large majority of costs can be considered variable. The analysis is based on the expected cost to public health funding bodies and care providers. Each of the unit cost estimates were reviewed and revised as appropriate by site teams (Table DS1). For example, all countries revised the costs of psychotropic medications from the default international rates set in the mhGAP costing tool, as well as salary amounts; South Africa revised upwards the unit costs of primary, secondary and tertiary hospital care utilisation.

### Coverage

Coverage refers to the proportion of people in need that receive a specified intervention. Thus, a target coverage rate of 50% for psychosis would mean that a half of all individuals with the condition in the population are expected by the final year of scale up to be actually accessing and receiving an intervention (for example, basic psychosocial treatment plus antipsychotic medication). Current coverage levels for each intervention were derived for each site based on estimates of prevailing service provision from local clinicians and national survey data; in many cases current rates of treatment coverage are extremely low (Table 2). For target coverage, estimates were based on the considered opinion of local research teams, taking into account expected levels of political, financial and logistical support for mental health service scale up (Table 2).

### Analysis

Site-specific output from the mhGAP costing tool was analysed in order to calculate costs per case and per capita for each disorder. Calculations were then made of selected relevant outputs such as total costs and incremental cost for packages of care and the proportion of total costs spent on different cost categories. A sensitivity analysis was also carried out on key cost drivers, including in-patient costs, scale-up patterns and the inclusion of inflation.

## Results

### Costs per case of disorder

The expected average annual cost of treating the different priority disorders in the non-specialist healthcare setting of each participating district is shown in Table 3. These are broken down by specific intervention components as set out in the WHO mhGAP intervention guide (for example basic psychosocial treatment, advice and follow-up; medication; intensive psychosocial treatments). In each of the five districts/countries, costs are highest for psychosis – reflecting the need for frequent follow-up visits as well as daily medication – and are particularly elevated in Nepal and South Africa (driven by antipsychotic drug costs). Costs are lowest for alcohol use disorders, reflecting lower resource utilisation (for example, brief advice or counselling). Costs per case in Ethiopia, India and Uganda are very comparable, and considerably lower than in South Africa.

### Resource needs and costs of scale up

**Fig. 1:**
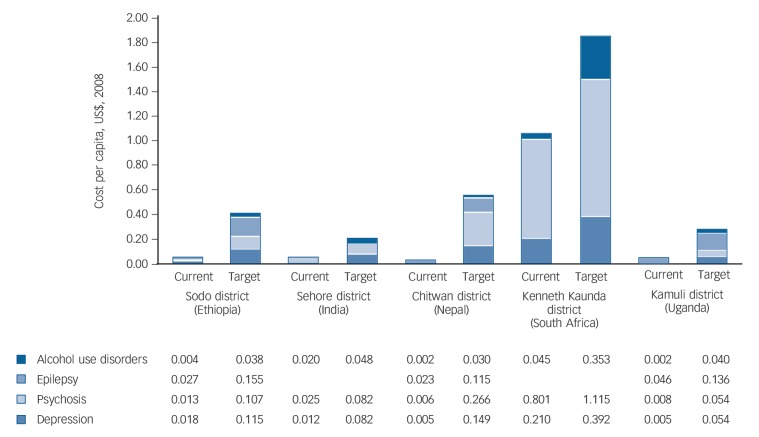
Cost of mental healthcare plan package, at current and target coverage (US$ per capita).

Multiplying the above costs per average case of disorder by the population in need (Table 1) and the proportion receiving treatment in the baseline year (Table 2) gives the total cost of the intervention package at current coverage levels. For example, the current cost of treating psychosis with antipsychotic medication and intensive psychosocial intervention in Sehore district in India is estimated to be $28 261 (4679 cases×20% coverage×$30.20 per case treated). For all disorders included in each district's mental healthcare plans, estimates of current cost ranged from $10 000 to 20 000 in the Ethiopian and Nepalese districts to over $700 000 in the South African district, equivalent to less than $0.10 to just over $1.00 per capita when differences in district population size are taken into account (Fig. 1). The total cost of delivering the intervention package at target coverage levels is naturally higher, ranging from $0.21 per capita in the Indian district (60% higher than current costs) to $1.86 per capita in South Africa (nearly 100% higher). Reflecting large changes in coverage, Nepal had the greatest relative cost increase (over threefold, from $0.04 to $0.56 per capita). In Ethiopia and Uganda, the total cost at target coverage levels is projected to reach $0.28–0.42 per capita.

**Fig. 2:**
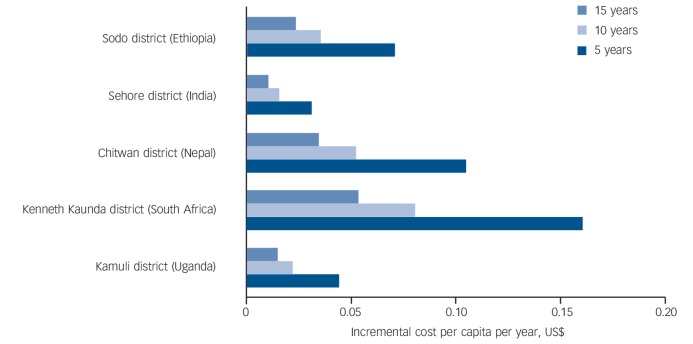
Incremental investment needed per year to reach target coverage levels over different scale-up periods (US$ per capita).

The difference between the current and target levels of treatment coverage gives the incremental cost of scaling up. The amount of funding that needs to be invested per person per year in order to move from current to target coverage levels depends on the period of scale up (the longer the period, the less that is needed per year). Figure 2 provides three time frames (5, 10 and 15 years) and their associated annual incremental cost values. Results indicate that, over a 10-year scale-up period for example, the additional amount that needs to be invested each year in all of the country settings is less than $0.10 per capita. In the context of Kamuli district in Uganda, for instance, the budget would need to increase each year over the next decade by about $9500 (an extra $0.02 per year for each of the 428 500 people living in the district); if the aim were to achieve set target coverage levels within the next 5 years, the annual budget increase would need to double to $19 000. For these analyses, a smooth linear pattern of scale up is employed for all settings, but depending on the extent of local or national stakeholder buy-in and logistical preparedness, the pattern could very well take on either a front-loaded or a slower, exponential trajectory.

**Fig. 3:**
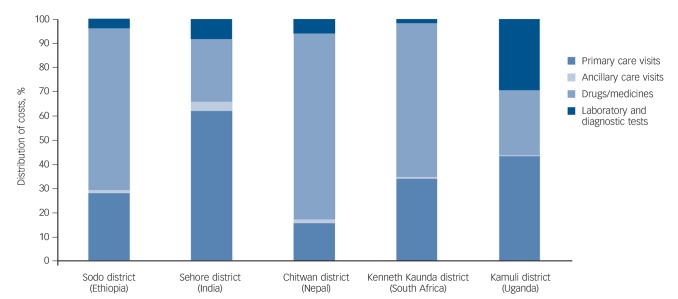
Distribution of costs in non-specialised healthcare settings.

Figure 3 provides a breakdown by category of cost in each of the five districts. Ancillary care and diagnostic tests account for only a small fraction; in the Ethiopian, Nepalese and South African districts, at least half of the costs are attributable to essential psychotropic medicines, with the remaining costs related to primary care service delivery, in particular human resources.

Based on the total volume of expected visits and the working conditions of healthcare providers, the mhGAP costing tool also produces estimates of human resource need. Table 4 shows the type and number of healthcare providers needed to deliver the specified mental healthcare intervention package in each of the districts at target levels of coverage. The estimated number of full-time equivalent staff needed per 100 000 population ranges from four to five in the Indian and South African districts to nearly ten in the Ethiopian district; inter-site variations are because of differences in the specified intervention package to be provided (for example inclusion of epilepsy in the MHCP of Uganda and Ethiopia) and associated coverage levels or resource inputs (for example lower target coverage levels for psychosis and epilepsy in Nepal than in Ethiopia and Uganda).

**Table 4:** Human resource needs at target coverage levels of intervention package delivery in five non-specialised healthcare settings

	Target full-time equivalents per 100000 population				
Human resource category	Sodo district (Ethiopia)	Sehore district (India)	Chitwan district (Nepal)	Kenneth Kaunda district (South Africa)	Kamuli district (Uganda)
Psychiatrist	0.0	0.2	0.3	0.2	0.0

Other physician/doctor	1.1	0.3	1.0	0.6	0.9

Nurse	4.2	1.9	2.5	1.7	4.7

Psychologist	0.0	0.3	0.3	0.3	0.0

Other psychosocial workers	2.1	0.6	1.3	0.8	0.9

Other providers/workers^a^	2.1	0.9	1.2	1.1	1.4

Total	9.5	4.2	6.6	4.7	7.9

a. Includes community health workers.

### Secondary and sensitivity analyses

**Fig. 4:**
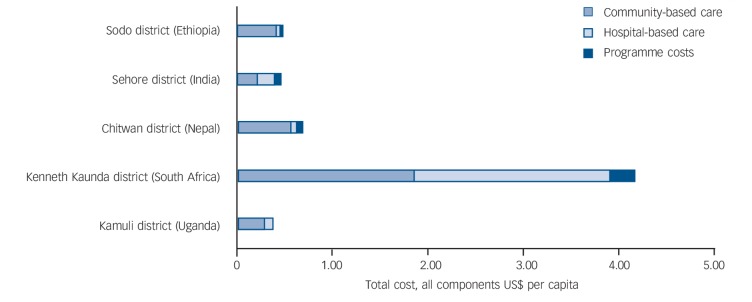
Sensitivity analysis: influence of hospital-based service use and programme costs on baseline results.

In line with the mhGAP intervention guide, but also reflecting the very limited availability of specialist service in the five districts, the baseline analysis presented above focuses on the care and treatment of patients in non-specialist healthcare settings alone; it does not include the costs associated with more specialist hospital-based services that some individuals access and use, nor does it include costs incurred above the provider facility level, such as programme management, training and supervision. Accordingly, further analysis was performed that included these additional costs, which would be incurred if specialist services are made available locally (in district general hospitals), or if MHCP implementation leads to new referrals of individuals with more severe conditions by non-specialist providers. Based on each country team's review of expected resource needs this included hospital admission for a proportion of individuals (2% for depression and alcohol use disorders, 5–10% with psychosis), periodic out-patient visits (20–30% for psychosis, 15–20% for other disorders) and the roll-out of mhGAP training programmes. Results for this expanded cost scenario showed that programme costs associated with training, supervision and management added 5–15% to the baseline cost estimate, except in India where the relative contribution is higher (28%). Incorporation of hospital-based services added a similarly modest amount (8–13%) to the baseline costs of the Ethiopian, Nepalese and Ugandan districts (reflecting the low unit costs of providing these specialist services in those settings) but increase costs in the Indian and South African districts (nearly and more than doubling the final cost, respectively). Figure 4 shows the revised costs of achieving target coverage levels with these components added. A sensitivity analysis was also undertaken to assess the impact of inflation (baseline results are in constant values for the year 2008). Over a 15-year-period of scale up, an inflation rate of 3% will increase final year costs by 50%, whereas a 6% inflation rate will more than double them.

## Discussion

### Main findings

The cost analysis presented here was carried out to inform local PRIME country teams about the expected resource implications associated with the implementation of their respective MHCPs. Such an analysis offers a complementary perspective to the (largely qualitative) formative research that went into the development of each site's plan;^5–9^ it produces information about the financial feasibility of these plans and can provide a ‘reality check’ where plans exceed what is likely to be possible or available in the future. Results indicate that, starting from an often very low base of mental health service coverage, the cost of scaled-up provision in non-specialist healthcare settings of an evidence-based package of care range from US$0.20 to 0.56 per capita in four out of the five districts assessed. For a district with a total population of half a million, therefore, an annual outlay of between US$100 000 and 300 000 would be required to reach the target coverage levels specified here. The outlier is South Africa, an upper-middle-income country where the prevailing price and quantity of healthcare service inputs are that much higher (to illustrate, total health spending per capita exceeds US$400 per year, compared with less than US$40 in the other four countries); the cost per capita of delivering the specified care package at target coverage levels in the South African district approaches US$2 per capita. This is higher than in the other countries but relatively low in the context of current health spending levels in South Africa. As indicated in the South African MHCP,^8^ mental health is incorporated within integrated chronic disease management at primary care level. Given that the additional cost of scaled-up provision will have to compete with other priority chronic conditions, the need for cost–benefit studies on the impact of integration on improved overall outcomes for chronic care is highlighted in the case of South Africa.

Getting to target levels of annual spending in each district will necessitate a steady budgetary increase, estimated at no more than US$10 000 extra per year for a district with half a million people if a 10-year period is used (even in the South African setting, since current coverage levels are relatively higher than in the districts of the other four sites). Extending the cost estimation to also take into account programme management and utilisation of hospital-based services by the district population in need increases these baseline cost projections, substantially so in South Africa and India (by as much as 100%), and modestly so in the other three sites (by approximately 20%). These upper cost estimates amount to only 1% of total current health spending per capita in the South African context, but reach up to 7% in Ethiopia.

Even with these additional cost elements of specialist care, programme management and training factored in, the present cost estimates are lower than those reported earlier for a similar mental healthcare package in a number of LMICs (including Ethiopia and Nepal), which suggested an indicative investment of US$2.00 per capita for low-income countries.^2^ This reflects the use of (and pragmatic need for) a more selective range of interventions and less intensive resource utilisation per treated case. In this sense, the current work provides a more realistic or locally grounded estimate of what can be achieved, and at what cost. Such a selective approach was also used recently in the context of the prevention and control of non-communicable diseases, which showed that the scaling up of a set of so-called ‘best buys’ would be expected to cost below US$1 in low-income countries, less than US$1.50 in lower-middle-income countries and US$2.50 in upper-middle-income countries.^11^

### Key cost drivers

Underlying these summary cost measures of programme needs and gaps, there are a number of key cost drivers at work in the different country contexts. One relates to local drug prices, which help to keep acquisition costs down in India but accounts for a large share of total cost in neighbouring Nepal as well as South Africa. Accordingly, where prices of essential psychotropic medicines represents a large share of spending, this could be a particular issue to investigate. A further critical category of cost concerns human resources and the specific type of worker that will undertake particular mental healthcare tasks or duties. For example, the community-based service model envisaged for scale up in Ethiopia assumes that no psychiatrist will be available within the district and that health extension workers will form the backbone of service provision. Therefore, appropriate policies and processes for the successful recruitment, training and retention of community-based mental health workers can be expected to have a major bearing on financial flows over time. Finally, the finding that costs increase most in South Africa and India when specialist services were included shows how hospital-based in-patient and out-patient care exerts an increasingly important influence on overall costs as national income rises. In low-income countries, an in-patient day often costs less than $10, and therefore has less impact on total costs; in high-income countries, by contrast, where one in-patient day typically costs hundreds of dollars, this is a critical driver of cost. These sources of variation between different national settings points to the need for context-specific data and analysis, which is very much what the mhGAP costing tool (available from the authors on request) is geared towards.

Such differences in the structure of costs make comparison between studies very hard, particularly between high- v. low- or middle-income countries. Nevertheless, other economic studies provide insights into average treatment costs for treating mental disorders in a range of settings. Using older antidepressant drugs and providing stepped care tailored to the needs of the patient, for example, has relatively low annual costs per case of depression, from US$107 in India to under US$200 in Nigeria.^12,14^ Similarly, the annual cost per treated case of epilepsy is relatively low; for example in Nigeria, older anti-epileptic drugs are less than US$100 per patient per year.^14^ In line with the evidence presented here, schizophrenia is generally more expensive to treat per person than either depression or epilepsy, especially if newer antipsychotic medications are used. In Nigeria, treating schizophrenia with older antipsychotic drugs falls between US$200 and 300, whereas newer antipsychotic drugs were estimated to cost over US$6000 per year.^14^ Similarly in Brazil, treatment with older, first-generation antipsychotic drugs is as low as US$120 per patient per year, but second-generation drugs cost over US$4000 per person annually.^15^ In Thailand, direct medical costs for drug treatment in combination with family interventions cost US$764 per patient per year.^16^

### Limitations

It is important to note that the costing approach and underlying tool used for this analysis does not provide any information on the impact of scaled-up investment on health or other outcomes resulting from MHCP implementation. Prospective assessment of health and economic outcomes forms an integral part of ongoing cohort studies being carried out in each PRIME site, and results from these studies will likely necessitate a refinement of the intervention care package elements and costs examined here; a comparison of planned *v*. actual expenditures is planned. Similarly, it will only be possible to compare the actual *v*. predicted deployment of human resources once district-level MHCPs have been fully implemented; until that time, estimates provided here rely on the need for and level of care indicated by each district's MHCP, as well as the existing supply and distribution of health workers at district level.

The costing tool used to generate the presented findings has been designed to provide for extensive adaptation and contextualisation (of selected disorders and interventions as well as their resource inputs and expected coverage), while offering a structured framework for putting together costed packages of care. Such flexibility is not only important for reflecting variations in service contexts or inputs between countries, but also within countries, for example where there is a highly decentralised form of government. However, the costing tool is unable to take proper account of critical health system constraints to service scale up, such as mid-term expenditure caps, supply-side bottlenecks to recruiting staff or accessing essential medicines, and inadequate referral and supervision mechanisms; such constraints have the capacity to radically alter the actual level of programme implementation or achievement. Even if such supply-side factors were managed successfully, there is the additional concern that demand for and actual uptake of available services does not match desired levels of effective coverage. These deficiencies are to be addressed via the development of a mental health module within the inter-United Nations OneHealth tool, which will enable more integrated planning by bringing together programme-specific needs for mental health and other disease programmes with shared health system components (such as human resource planning and budgeting).
